# 

**Published:** 1903-12-05

**Authors:** 


					THE hospital.   "The standard of highest purity."?Lancet. December 5th, 1903.
smasw r cad.^.ry's
"A PfRFEJT .j^OOD."
COCOA
NO DRUQS OR ALKALIB8 DUD>
No. 897. Vol. XXXV. [Kfi5?p?] Saturday, Dec. 5th, 1903. [Sub^ptp?64Mm?'] PRICE THREEPENCE.

				

